# Assessing physical literacy and establishing normative reference curves for 8–12-year-old children from South Punjab, Pakistan: The PAK-IPPL cross-sectional study

**DOI:** 10.1371/journal.pone.0312916

**Published:** 2025-02-11

**Authors:** Syed Ghufran Hadier, Liu Yinghai, Liu Long, Syed Danish Hamdani, Syed Muhammad Zeeshan Haider Hamdani

**Affiliations:** 1 School of Physical Education, Shanxi University, Taiyuan, Shanxi Province, China; 2 Department of Sports Sciences, Bahauddin Zakariya University, Multan, Punjab, Pakistan; 3 School of Physical Education, Suzhou University, Suzhou, Anhui, China; 4 Division of Olympic Sports, China Swimming College, Beijing Sport University, Beijing, China; 5 School Education Department, Government of Punjab, Multan, Pakistan; 6 Faculty of Social Science, Department of Sports Sciences, Bahauddin Zakariya University, Multan, Punjab, Pakistan; Southwest University, CHINA

## Abstract

**Background:**

Physical literacy (PL) encompasses an individual’s motivation, confidence, physical competence, and knowledge, all of which foster lifelong engagement in physical activities. In developing countries like Pakistan, there is a pressing need to assess PL status using culturally valid tools. This study aims to evaluate PL among children aged 8–12 in South Punjab, Pakistan while developing normative reference curves and exploring factors influencing PL.

**Methods:**

A cross-sectional study was conducted with 1,360 students (mean age = 10.00, SD = 1.41 years) from 85 schools, using a culturally adapted and validated version of the CAPL-2 tool. The CAPL-2 scoring system was validated to align with the population and cultural context. Statistical analyses, including t-tests, Chi-squared tests, Pearson’s correlation, multivariate logistic regression, and Classification and regression tree methods, were performed to identify factors associated with PL classifications, with significance set at p < 0.05. The Generalized Additive Models for Location, Scale, and Shape were used to create age- and sex-specific PL normative reference curves.

**Result:**

The study revealed significant influences of gender, socioeconomic status (SES), and locality on PL. Boys consistently scored higher in PL across all domains compared to girls, with significant differences (p < 0.001) in all but the knowledge and understanding domain (p = 0.160). Boys’ PL scores were 6% higher overall, with a 7% and 5% advantage in the daily behavior and physical competence domains, respectively. Children from low SES and rural settings exhibited significantly higher PL scores than their middle/high SES and urban counterparts (p < 0.001). Walking to school was positively correlated with higher PL scores, while children traveling by car had the lowest scores (p < 0.001). Anthropometric differences between genders were noted, further emphasizing the disparities in physical competence. Most participants (71.6%) fell into the "Beginning" or "Progressing" PL categories, with females predominantly in these lower stages.

**Conclusion:**

This study reveals that gender, SES, and locality significantly impact PL among children in South Punjab, Pakistan. Boys, lower SES groups, and rural children showed higher PL scores, with everyday activities like walking to school positively contributing to PL development. These findings emphasize the need for targeted, demographically sensitive interventions to enhance PL in the region.

## 1 Background

Physical activity (PA) is crucial for children’s physical and mental well-being. The World Health Organization (WHO) recommends that children and adolescents aged 5 to 17 engage in at least 60 minutes of moderate to vigorous physical activity (MVPA) daily [1]. This recommendation addresses concerns about decreasing PA, increasing screen time, childhood obesity, high blood pressure, and increased mortality [2]. Numerous countries are actively promoting physical activity, including a collaboration among 25 European countries analyzing data on obesity and advocating regular physical activity as a preventive measure across all ages, genders, ethnicities, and socioeconomic subgroups [3].

Despite these efforts, studies show that over half of children aged 6 to 11 fail to meet recommended physical activity levels [4]. This sedentary lifestyle established in childhood often persists into adolescence and adulthood [5]. Consequently, physical literacy (PL) has been recognized as pivotal for PA and sports, supporting holistic development, especially when nurtured in early childhood [6]. Physical literacy encompasses motivation, confidence, physical competence, knowledge, and understanding to value and take responsibility for lifelong engagement in physical activities [6], as defined by the International Physical Literacy Association [7]. It holds particular significance in early childhood, providing a foundation for overall development [8].

Differences in PL among children can be influenced by cultural, social, and physical environments [9–11], and understanding these variations can contribute to more effective interventions for children’s health and well-being. Recent research highlights the potential of PL in establishing active lifestyle habits and promoting holistic development during late childhood, a critical period when PA levels decline [12, 13]. Considering the numerous benefits of PA, including academic achievement, PL has the potential to contribute to education, sports, PA, recreation, and public health [14]. Consequently, organizations worldwide have embraced PL and implemented interventions for children in education and sports [15]. The Canadian Assessment of Physical Literacy (CAPL) is an established measure used in multiple countries to assess children’s capacity for leading a physically active lifestyle [16].

Given the dynamic nature of PL, there is a demand for its assessment to facilitate monitoring, surveillance, and program evaluation [5]. However, the current understanding of children’s Physical Literacy levels worldwide is limited, necessitating further research on PL development across ages, genders, cultures, and pedagogical contexts [5]. Cultural contexts and social and physical environments can influence PL levels [9, 10, 16], yet only a few studies have examined different cultural settings [16, 17]. Therefore, additional research is imperative to gain insights into PL development across different contexts and demographics.

This study aims to establish the baseline status of Physical Literacy among children aged 8 to 12 in Pakistan, stratified by sex, age, and sociodemographic factors. In Pakistan, low levels of physical activity and a high prevalence of childhood obesity underscore the importance of exploring PL [18–20]. Understanding PL in this context is vital for developing policies that promote physical activity and health at both the individual and community levels.

## 2 Methods

### 2.1 Study design and sampling

This cross-sectional study is part of the Pakistan Initiative to Promote Physical Literacy (PAK-IPPL). A stratified random sampling method was employed to divide the South Punjab province into three strata: Multan, Bahawalpur, and Dera Ghazi Khan, ensuring the representation of geographical and demographic variations within the province [18, 21]. The sample size was calculated using Cochran’s formula: $n=\frac{{\left(Z\right)}^{2}PQ}{e^{2}}\times D$ [22, 23]. In this formula, Z represents the standard normal distribution, which is 1.96 at a 5% significance level. The expected proportion (P) is 0.23, while Q is 1—P (0.766), the level of precision e2 is 0.002545, and D, the design effect, is set to 5 [24]. This resulted in a calculated sample size of approximately 1360 participants. To ensure regional representation, 87 higher secondary schools were chosen from the three divisions: Multan, Bahawalpur, and Dera Ghazi Khan, with 29 schools per strata using equal allocation method [25]. However, two schools from the Bahawalpur and Dera Ghazi Khan divisions declined participation, resulting in 85 schools being included in the study.

A list of students aged 8–12 was obtained from each school, and 16 students were randomly selected. During field testing, 11% (150 students) either withdrew or were unable to complete the CAPL-2 protocol. To maintain the sample size as determined by the sample size calculation, an additional random sample was taken to compensate for the 11% non-response rate. This process was repeated until a 100% response rate was achieved. The final sample consisted of 455 participants from both Multan and Bahawalpur and 450 from Dera Ghazi Khan, ensuring balanced representation across schools, ages, genders, and cities, as reported in a previous study [24, 26]. Moreover, the children’s enthusiasm for learning and performing new tasks, as well as their desire to compete with their peers, played a significant role in motivating them to participate in their studies. Additionally using pedometers to assess daily activity levels increased the children’s interest in completing the task, which led to a high response rate for the completion of the study.

### 2.2 Ethics board approval

The study was approved by the Ethics Board of the School of Physical Education at Shanxi University, China (Approval No: SXULL2019012), with approval dates on 2 January 2019 and 3 July 2023, in accordance with the Declaration of Helsinki. To ensure compliance with local regulations in Pakistan, additional approval was obtained from Bahauddin Zakariya University, Multan (Letter No: 374/UREC/2020), along with permission from the South Punjab Education Department (Letter No: 2189/GB).

### 2.3 Procedures and measures

This study was conducted during the 2020–2021 academic year using established questionnaires to gather demographic and anthropometric data alongside valid and reliable tools to assess physical literacy and its domains [24, 26].

A standardized schedule was followed for data collection across various schools. Each school was allocated three days to complete the assessments, ensuring consistency and efficiency. The researcher visited multiple cities, maintaining this standardized approach across all locations. On the first day, students were briefed on the study’s purpose and introduced to the CAPL-2 tool. The research team answered questions before distributing a self-designed questionnaire and the CAPL-2U (Canadian Assessment of Physical Literacy 2nd edition-Urdu version) [27]. A team member was available to assist participants with comprehension or completion issues.

On the second day, the Canadian Agility and Motor Skill Assessment (CAMSA) and plank test were completed under the supervision of 5 appraisers. Before the assessment, an assessor demonstrated and instructed the students on how to perform the test, and each student was given one practice trial for the plank and two practice trials for the CAMSA before the actual evaluation. On the third day, anthropometric data and the PACER test were completed, and the pedometer and its instructions and tracking log sheet were given to the participants. Missing pedometer day data were computed using the procedures described in the CAPL-2 manual [28]. If a participant could not attend school on the assessment day, we evaluated the missing test the next day (since the data collection team visited each school at least four times), or the date for test evaluation was on the pedometer return day.

**Fig 1:**
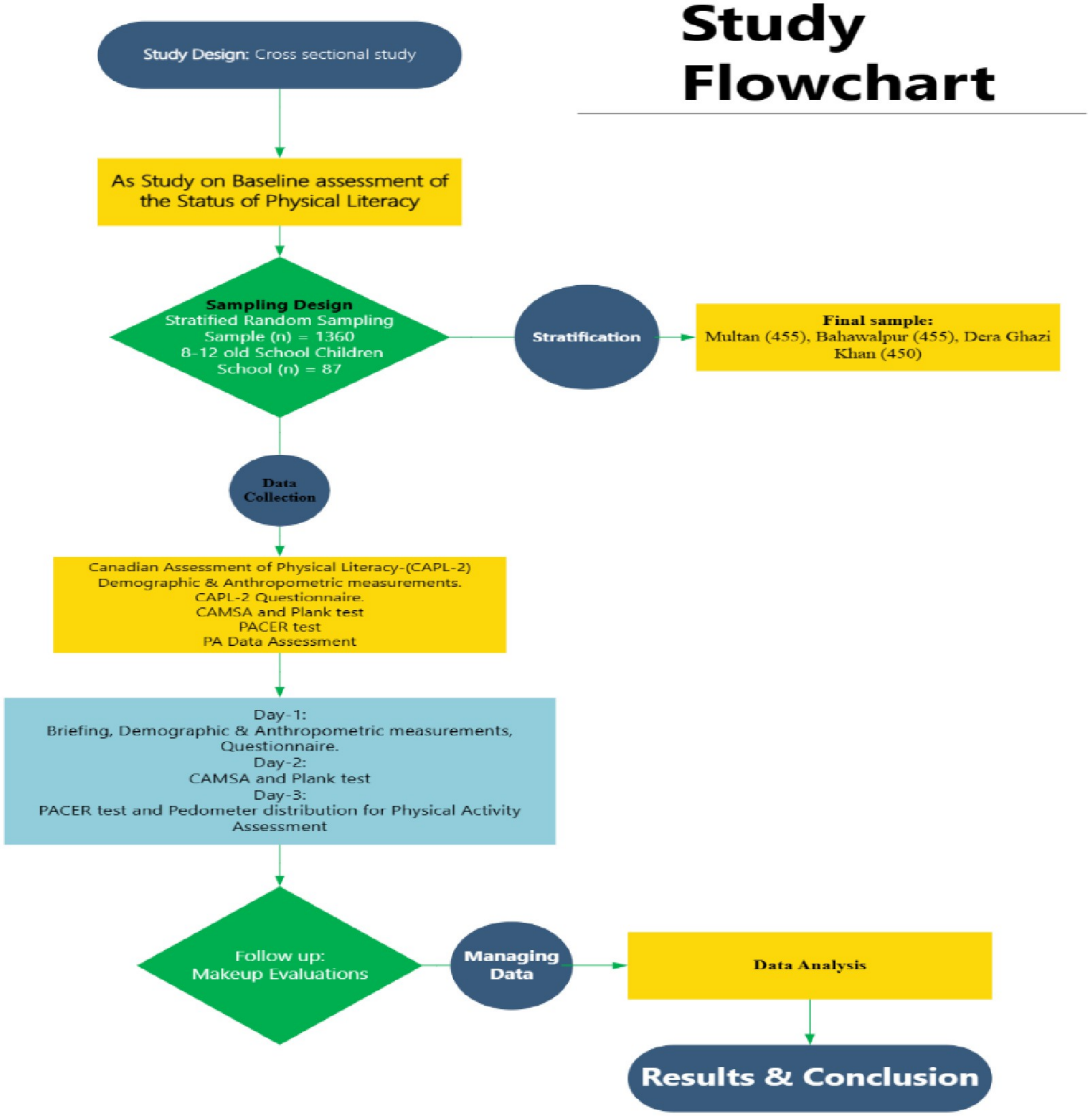
Study flow chart.

As the study involved children aged 8–12, parental or guardian consent, either written or verbal, was obtained for all participants. Data collection and testing were conducted during drill (physical education) classes, to avoid interference with the teaching schedule. Students were only permitted to participate in the study if they were able to carry out everyday activities normally, were psychologically healthy, had no evident physical defects, could correctly comprehend the test requirements, and were willing to comply with the completion of the tests. Fig 1 shows the process of study. Approval of using the CAPL-2 questionnaire and related materials was obtained from the HALO Research Group prior to data collection.

#### 2.3.1. Demographics and anthropometrics

The students’ ages and birth years were obtained from the school record. Height was measured using a Digital Electronic Height-Weight Measurement Scale. Students were instructed to maintain a straight posture while standing barefoot against a level surface with their backs against the wall. Weight was measured in kilograms (kg) using the same electronic scale, calibrated for accuracy. Participants removed shoes, excess clothing, and accessories. The weight reading, precise to 0.1 kg, was recorded when stable for three seconds.

Body mass index (BMI) was calculated using the CDC-endorsed standard formula: $BMI=\frac{W(kg)}{{Hm}^{\left(2\right)}}$ for BMI calculation and assessment [29]. The CDC percentile classifications were used to classify weight statuses. The waist circumference was measured using a standard tape roughly 1 centimeter above the navel, with measurements recorded in centimeters to one decimal place. Hip circumference was measured using standard tape at the broadest part over the buttocks. The Waist-Hip Ratio (WHR) was derived by dividing waist circumference by hip circumference. This ratio, derived from dividing waist circumference by hip circumference, reflects central adiposity [30].

This study used the Body Adiposity Index (BAI) to estimate body fat percentage in children based on height and hip circumference. Costly techniques like dual X-ray absorptiometry (DXA) and air displacement plethysmography (ADP) were deemed unsuitable for the pediatric population [31]. The given formula, $BAI=\frac{Hip\;(cm)}{{Height\;\left(m\right)}^{1.5}}-18$, was employed in this study for the calculation of BAI [32]. provided a valid and cost-effective method for calculating body fat percentage. The Relative Fat Mass (RFM) approach was employed to assess whole-body fat percentage, using the RFM pediatric equation incorporating variables such as height, waist circumference, and sex. The following equation was used *RFMp* = 72 − (22 × *height* ÷ *waist circumference*) + (5 × *sex*) [33]. In the equation the sex equals 0 for boys and 1 for girls.

#### 2.3.2. Physical literacy assessment

**Fig 2:**
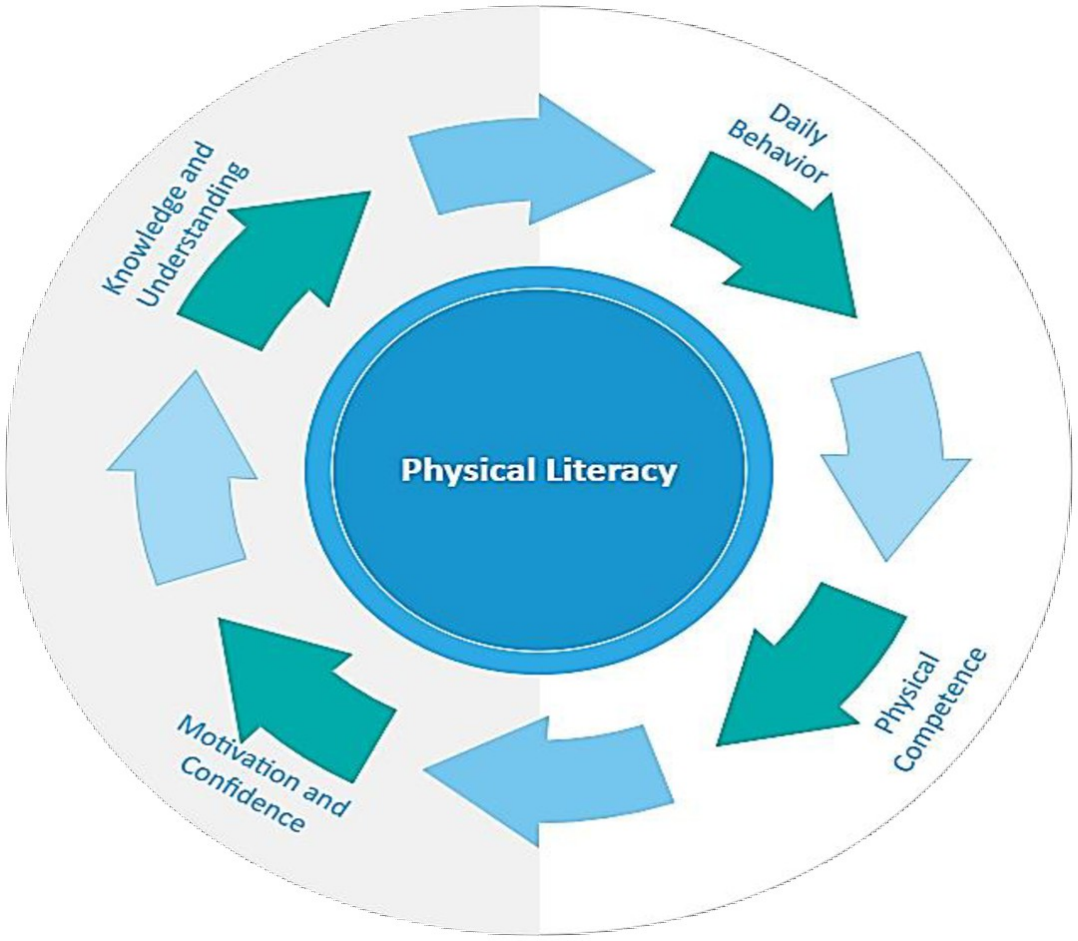
The core domains of physical literacy.

The study utilized the Canadian Assessment of Physical Literacy- second edition (CAPL-2) to assess children’s physical literacy and domains levels (Fig 2). CAPL-2 is a holistic tool that evaluates fundamental movement skills, physical fitness, knowledge, and motivation toward physical activities. It employs a multi-dimensional approach to assess overall physical capabilities and engagement, recognizing the importance of daily behaviors in promoting physical literacy. The CAPL-2 domains can be used independently to provide scores for each component or collectively to obtain children’s comprehensive physical literacy status.

*Physical Competence (PC)*. This domain encompasses cardiovascular endurance, muscular strength, and motor skills. Composite scores of 30 were derived from three evaluations.

***Cardiovascular Endurance***: the 20m shuttle run test, also known as the Progressive Aerobic Cardiovascular Endurance Run (PACER) test, was administered to assess cardiovascular endurance. The PACER test required children to run back and forth between points at an increasing pace. It was awarded ten domain points and scored 1 to 10 based on completed laps as per CAPL-2 scoring. ***Muscular Strength***: The Plank Test in CAPL-2 measured the muscular strength generated by muscles or muscle groups. Children held a push-up position to assess core and upper body strength. Scores ranged from 1 to 10 according to duration. ***Agility and Motor Skills***: The Canadian agility and motor skill assessment evaluated fundamental movement skills, including running, jumping, throwing, catching, balancing, and coordination. CAMSA employed structured activities and observational assessments for agility, balance, coordination, and reaction time. Children’s performance was ranked 1 to 10 based on prescribed criteria in the manual.

*Knowledge and Understanding (K&U)*. This domain assesses children’s knowledge and understanding of recommended physical activity levels, the benefits of PA, and methods to improve cardiorespiratory and muscular endurance, as well as sports skills. Children were asked to complete four questions based on four multiple-choice questions, each worth one point, and one fill-in-the-blanks passage to evaluate knowledge about improving specific components of physical competence, worth six points. Overall, this domain has a total of ten points in the overall PL scores.

*Motivation and Confidence (M&C)*. This domain consists of a 12-item questionnaire designed to assess children’s motivation and self-confidence towards physical activity, divided into four sub-constructs: (a) Intrinsic Motivation: Evaluates children’s natural desire to engage in physical activity, including attitudes, beliefs, emotions, self-confidence, and enjoyment. (b) PA Self-Competence: Measures the belief in one’s ability to successfully participate in physical activities, focusing on self-confidence, competence, and resilience. (c) Predilection: Assesses preferences for different types of physical activities based on interests and tendencies. (d) Adequacy: Evaluates one’s perception of their ability to meet physical activity demands and perform effectively. Each item in this section of the questionnaire scores 2.5, with a maximum domain score of 30, providing insights into children’s motivation, confidence, and attitudes toward physical activity.

*Daily Behavior (DB)*. This domain evaluates physical activity and MVPA through both objective and subjective measures. Scores are categorized into four levels: Beginning, Progressing, Achieving, and Excelling, indicating whether children meet recommended activity levels.

*Objective method*. In this study, Yamax DigiWalker SW-200 pedometers (Digiwalker, Yamax Corporation, Tokyo, Japan) were used to measure PA. These pedometers were chosen for their high reliability, with a strong correlation to observer steps (ICC > 0.90) [34]. Pedometers were selected as a cost-effective and well-tolerated alternative to accelerometers and heart rate monitors used in previous studies [35]. While pedometers may not capture all activities, they have shown a strong correlation with step counts during walking and treadmill trials [36]. They have also been found to correlate with accelerometer counts, oxygen consumption, and heart rate [36]. Pedometers are suitable for assessing PA in children due to their objectivity, reliability, and validity [37]. Each child wore the pedometer for a minimum of 10 consecutive days, with an average step count over seven days representing their weekly PA. Scoring and cut points from CAPL-2 were used to determine MVPA, providing valuable information on children’s physical activity volume.

*Subjective method*. The subjective method involves children self-reporting their MVPA engagement in the previous seven days. MVPA refers to activities that raise the heart rate. Children chose the number of MVPA days and scored from 0 to 5. A score of 0 means no MVPA days, while 6 or 7 receives a score of 5. This method provides insights into children’s perception of MVPA engagement. In the Daily Behavior domain, objectively measured physical activity contributes 25 scores, while objectively measured MVPA contributes 5 scores. Together, they total 30 scores for calculating the PL score. This information helps understand individuals’ activity engagement and guides targeted interventions for promoting a healthy lifestyle.

*CAPL-2 scoring*. The CAPL-2 tool was used to assess physical literacy in children. Normative reference values were established using data from Pakistani children to ensure cultural relevance and accuracy. Age- and sex-specific percentiles were generated using Generalized Additive Models for Location, Scale, and Shape (GAMLSS), allowing for score comparisons across different groups. Domain scores and the overall PL score were classified into four categories (Table 1) [28]:

**Table 1:** The CAPL-2 score interpretation within four categories.

CAPL-2 score interpretation	Categories
< 17th percentile	Beginning
17th to 65th percentiles	Progressing
above the 65th percentile to the 85th percentile	Achieving
> 85th percentile	Excelling

1- ***Beginning***: Signifies insufficient PL levels, which implies that the children at this level are still in the initial stages of developing their physical literacy. 2- ***Progressing***: Indicates improvement in PL but without reaching an acceptable level. Children in this category are making strides but need further development to reach a sufficient level of physical literacy. 3- ***Achieving***: Represents a sufficient PL level. This is the minimum level that CAPL’s scoring system recommends, meaning that children at this stage have developed adequate skills, knowledge, and attitudes necessary for a physically active lifestyle. 4- ***Excelling***: Indicates the highest performance in physical literacy, representing children who not only meet the adequate levels but excel in physical skills, knowledge, and attitudes.

By categorizing the scores into these groups, stakeholders such as PE educators, parents, and policy makers can get a clear picture of where children stand in terms of physical literacy and can use this information to design interventions, programs, or strategies aimed at improving physical literacy among Pakistani children.

### 2.4 Statistical analysis

Data was analyzed using IBM SPSS version 22 and RStudio. Descriptive statistics characterized variables, including means, standard deviations, and percentages. Outliers were identified using Z-scores set at 5, and adjusted to maintain result reliability while preserving the sample size of 1,360 children. Data normality was assessed through Q-Q plots and histograms. Gender differences in various factors were examined using Independent Samples t-tests, and Chi-squared tests were employed to compare categorical characteristics among weight groups. Age- and sex-specific cut points and percentile curves were established using GAMLSS. MANOVA was utilized to analyze these disparities further. Linear relationships between BMI, overall PL, and DB domain scores were assessed using Pearson’s correlation. Odds ratios with 95% confidence intervals were calculated through multivariate logistic regression. To clarify interactive combinations of factors associated with physical literacy level classification, a Classification and regression tree (CRT) analysis was employed. Statistical significance was defined as p < 0.05.

## 3 Results

The study aimed to evaluate the physical literacy of school-aged children (8–12 years) in South Punjab, Pakistan, focusing on three densely populated districts: Multan, Bahawalpur, and D. G. Khan. These districts represented 67% (Multan and Bahawalpur with 910 children each, or 33.5% per district) and 33.1% (450 children in D. G. Khan) of the total study population. The sample comprised 1,360 participants, detailed in Table 2, which includes demographic information and descriptive statistics, segmented by age and gender. Among them, 76.8% (1,045 children) were from urban areas, while 23.2% (315 children) hailed from rural locales. The gender distribution was nearly equal, with 49.6% (675) boys and 50.4% (685) girls. Each grade, from the 4th to the 8th, had an approximate 20% representation, with boys and girls almost evenly distributed across grades.

**Table 2:** Age and gender-specific descriptive characteristics of the study participants.

Variable	Boys (*n* = 675) $\bar{\text{x}}\pm \text{SD}$	Girls (*n* = 685) $\bar{\text{x}}\pm \text{SD}$	*p*-Value
Height, (m)	137.79 ± 11.42	136.74 ± 11.25	0.088
Body weight (kg)	30.82 ± 8.82	30.34 ± 8.41	0.308
BMI (kg/m2)	16.04 ± 3.19	16.06 ± 3.14	0.920
***BMI Classes (CDC)***			
Underweight (<5th) %	32 (4.8)	37 (5.5)	-
Normal weight (≥5th—<85th) %	546 (80.9)	539 (78.7)	-
Overweight (≥85th—<95th) %	52 (7.8)	61 (8.9)	-
Obesity (≥95th) %	42 (6.2)	51 (7.4)	-
BMI, z scores	0.01±1.01	-0.01±0.99	0.654
WC (cm)	60.35 ± 9.40	58.10 ±7.89	<0.001
HC (cm)	64.12 ± 7.60	66.16 ± 8.66	<0.001
WHtR	0.95 ± 0.19	0.89 ± 0.13	<0.001
Body Fat % (BAI)	21.90 ± 5.46	23.17 ± 6.02	<0.001
Whole-Body Fat % (RMF)	27.49 ± 9.42	21.51 ± 7.76	<0.001
HG Strength	23.49 ± 7.23	19.40 ± 7.08	<0.001

**Note**: Data is presented as $\bar{\text{x}}$: mean; SD: standard deviation (or percentages, as indicated); BMI: Body mass index; WC: Waist circumference; HC: hip circumference; WHtR: Waist/hip circumference ratio; BAI: Body Adiposity Index; %BF: body fat percentage; RMF: Relative Fat Mass; HG Strength: Hand grip Strength; *p*-value significant at < 0.05.

The mean age of participants was 10.00 ± 1.41 years. Physical characteristics averaged to a height of 137.26 cm, a weight of 30.58 kg, and a BMI of 16.05 kg/m^2^. BMI categorization indicated that 5% were lean, 79.8% had a normal weight, 8.7% were overweight, and 7.1% obese. A higher percentage of boys fell into the normal weight category, while a greater proportion of girls (16.3%) were in the overweight or obese categories. Gender-specific anthropometric data showed mean BMI z-scores of 0.01 ± 0.99, waist circumferences of 59.22 ± 8.74 cm, hip circumferences of 65.15 ± 8.21 cm, waist-to-hip ratios of 0.92 ± 0.17, body fat percentages of 22.81 ± 8.82%, and whole-body fat percentages of 24.48 ± 9.14%. Notably, girls exhibited higher hip circumference, body, and whole-body fat percentages, whereas boys had greater waist circumference and waist-to-hip ratios. Additionally, the average hand grip strength was 21.43 ± 7.44, with boys displaying significantly higher strength than girls, highlighting gender differences in physical strength.

**Fig 3:**
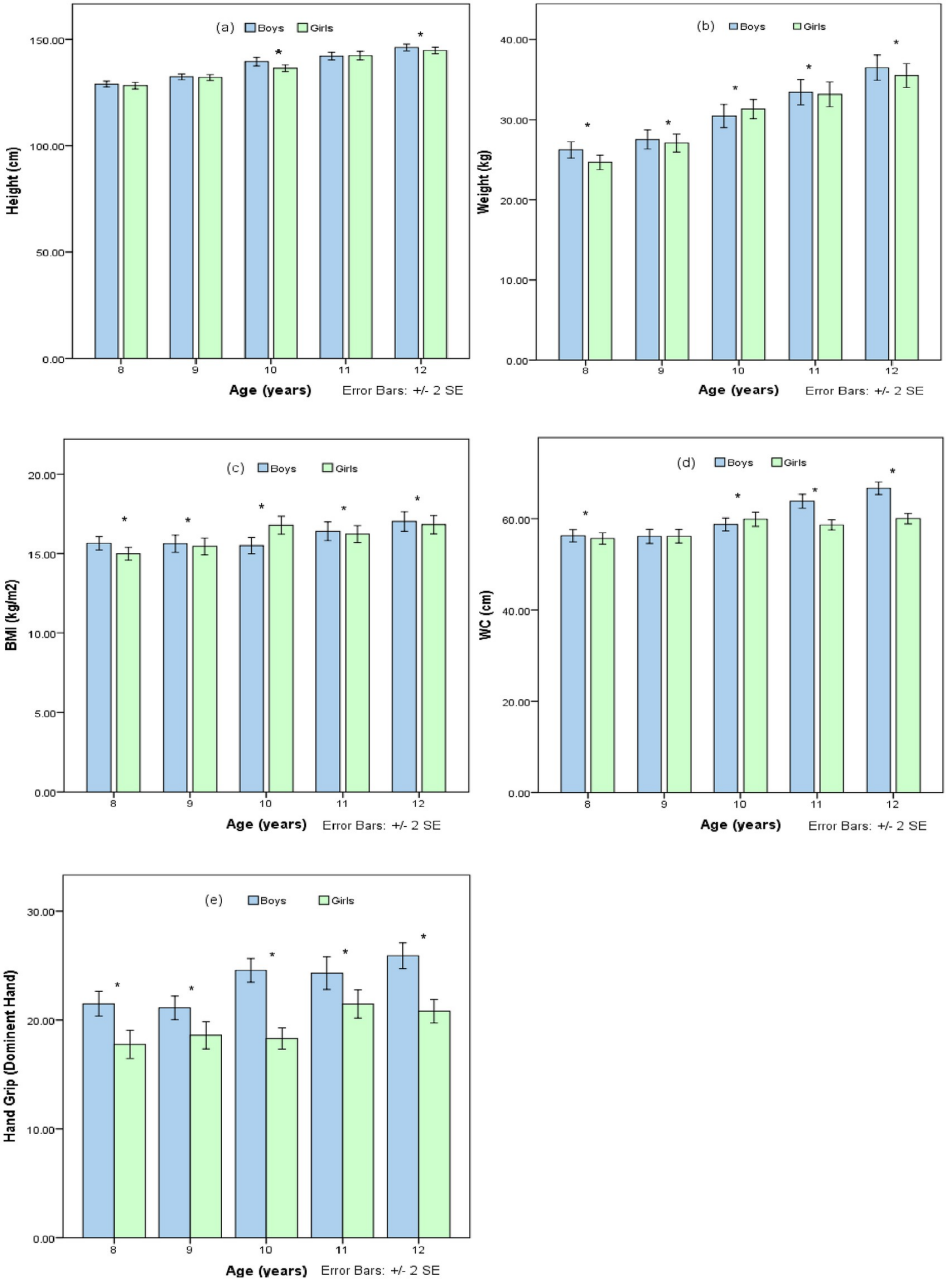
Age and gender-specific growth variables (a) Height, (b) Weight, (c) BMI, (d) WC, and (e) HG among children of South Punjab; *: Significant at < 0.05.

In exploring developmental trends, the Fig 3 bar graphs detail the progressive changes in anthropometric and physical strength attributes among boys and girls aged 8 to 12. Height assessments reveal a consistent growth trajectory in both genders. However, the analysis signifies that boys exhibit a superior height increment, with the disparity becoming statistically significant at the age of 10 and persisting thereafter. The weight parameter follows a similar incremental pattern, with boys showing a significantly higher weight from the age of 10, indicative of divergent growth rates between the sexes.

BMI trends diverge distinctly post age 9, where girls begin to exhibit a higher BMI. This variance attains statistical significance at ages 11 and 12, suggesting a gender-specific shift in body composition during these critical preadolescent years. Waist circumference, a marker of central adiposity, aligns with BMI findings, with girls demonstrating a higher measurement from age 9, and these differences reaching significance at multiple age points. Handgrip strength, an alternate for muscular strength, shows boys outperforming girls throughout the age spectrum, with significant differences materializing from the age of 10. This trend amplifies with age, reinforcing the notion of gender-based differences in muscle strength development. The data collectively underscores the developmental discrepancies between boys and girls, highlighting the necessity of considering gender-specific strategies in health and physical education during these formative years.

Table 3 displays the descriptive statistics for the children’s Socioeconomic Status, school traveling mode, and household size. Of the participants, 906 (66.61%) were in the low SES category (501 boys, 405 girls), 269 (19.19%) in the middle SES, and 193 (14.19%) in the high SES, with a significant gender-SES association (p<0.001). For STM, walking was most common (472 participants; 264 boys, 208 girls), followed by motorbike (430; 230 boys, 200 girls), bicycle (214; 105 boys, 109 girls), bus (177), and personal cars (67), with a significant gender-STM link (p<0.001). Regarding FM, 677 (49.77%) had ≤5 family members, with diminishing frequencies for larger household sizes and a notable gender-FM association (p<0.001).

**Table 3:** Descriptive characteristics of SES, STM & family member of South Punjab students of age 8–12.

Variable	Boys (n)	Girls (n)	Frequency (%)	*p*
**SES**				
Low SES	501	405	906 (66.61)	<0.001
Middle SES	129	132	261 (19.19)	
High SES	45	148	193 (14.19)	
**STM**				
By walk	264	208	472 (34.70)	<0.001
By Bicycle	105	109	214 (15.73)	
By Motorbike	230	200	430 (31.61)	
By School Bus	55	122	177 (13.01)	
By Personal Car	21	46	67 (4.92)	
**Family Member**				
≤3	255	164	389 (28.60)	<0.001
≤5	341	336	677 (49.77)	
≤8	88	156	244 (17.94)	
≤10	20	29	49 (3.60)	
≥10	0	1	1 (0.07)	

**Note**: SES: Socioeconomic Status; STM: school Transport mode; *P*-value < 0.001 means significant difference.

Table 4 shows gender-specific scores for Physical Literacy and its domains. Overall, the average physical literacy score was 51.06 out of 100, with boys scoring higher (53.57) than girls (48.58). In the daily behavior domain, boys averaged 12.89 and girls 11.47 out of 30. In physical competence, boys scored 17.39 and girls 14.53. For knowledge and understanding, the scores were closer, with boys at 6.44 and girls at 6.32 out of 10. In motivation and confidence, boys averaged 18.42 and girls 17.62. Boys generally scored higher than girls, with statistically significant differences (p < 0.001) in all but the knowledge and understanding domain, where the difference was not significant (p = 0.160).

**Table 4:** Gender-Specific composite scores for physical literacy and domains.

Variable	Total (n = 1360) Mean ± SD	Boys (n = 675) Mean ± SD	Girls (n = 685) Mean ± SD	P-value	Minimum & Maximum
**Total PL** (0–100 scores)	51.06±10.20	53.57±9.94	48.58±9.85	<0.001	23.33–84.02
**DB** (0–30 scores)	12.18±4.62	12.89±5.22	11.47±3.81	<0.001	4.95–26.79
**PC** (0–30 scores)	15.95±3.92	17.39±3.24	14.53±4.02	<0.001	3.35–25.58
**K&U** (0–10 scores)	6.38±1.60	6.44±1.55	6.32±1.63	.160	2.97–9.90
**M&C** (0–30 scores)	18.02±5.55	18.42±5.85	17.62±5.21	<0.05	6.53–29.69

**Note**: DB: Daily Behavior; PC: Physical Competence; K & U: Knowledge & Understanding; M & C: Motivation & Confidence; PL: Physical Literacy.

In Table 5, the results show gender-based differences in Physical Literacy domain scores. In the Daily Behavior domain, boys had a higher average daily step count (8136.48) than girls (7589.50) and higher levels of MVPA (boys: 5.17, girls: 4.79), with both differences being statistically significant (p < 0.001). Within the Physical Competence domain, boys outperformed girls in the CAMSA assessment (boys: 12.04, girls: 11.00), the Pacer (20m) test (boys: 19.90, girls: 17.22), and the Plank test (boys: 105.68, girls: 98.62), with all differences being statistically significant (p < 0.001). No significant gender differences were noted in the Knowledge and Understanding domain, with boys and girls showing similar mean scores (boys: 2.72 and 3.70, girls: 2.70 and 3.62, p > 0.05). In the Motivation and Confidence domain, boys scored higher than girls in three out of four components (predilection, adequacy, and intrinsic motivation), with significant differences (p < 0.05). However, no significant difference was observed in the physical competence scores between boys (4.44) and girls (4.34) (p > 0.237).

**Table 5:** Physical literacy domains specific scores stratified by sex.

Variable	Total (n = 1365) Mean ± SD	Boys (n = 675) Mean ± SD	Girls (n = 685) Mean ± SD	P-value
**DB** (30 points)				
Average Daily Steps	7860.98±2180.18	8136.48±2486.48	7589.50±1789.66	<0.001
Self-Reported MVPA	4.98±1.10	5.17±1.07	4.79±1.09	<0.001
**PC** (30 points)				
CAMSA (28 scores)	12.04±2.99	13.09±2.82	11.00±2.79	<0.001
PACER (20m)	19.90±8.04	22.61±7.59	17.22±7.57	<0.001
Plank (sec)	105.68±35.94	112.85±32.94	98.62±37.38	<0.001
**K & U** (10 points)				
Knowledge scores	2.72±0.60	2.72±0.56	2.70±0.63	0.185
Understanding scores	3.66±1.16	3.70±1.15	3.62±1.17	0.211
**M & C** (30 points)				
Predilection Score	4.54±1.90	4.66±1.92	4.43±1.87	<0.05
Adequacy Score	4.72±1.81	4.84±1.84	4.61±1.77	<0.05
Intrinsic Motivation	4.37±1.68	4.48±1.78	4.25±1.57	<0.05
Physical Competence	4.39±1.58	4.44±1.62	4.34±1.54	0.237

**Note**: DB: Daily Behavior; PC: Physical Competence; CAMSA: Canadian Agility and Movement Skill Assessment; PACER: Progressive Aerobic Cardiovascular Endurance Run; K&U: Knowledge & Understanding; M&C: Motivation & Confidence; PL: Physical Literacy.

Table 6 shows the Physical Literacy scores across socioeconomic status, school traveling mode, and residential areas. Participants mainly belonged to the Low SES group (n = 906), which had the highest PL score of 53.37 (SD = 10.06). Their scores were higher in all domains except Knowledge and Understanding, where they scored 6.37. Middle SES (n = 261) and High SES (n = 193) groups had similar average PL scores of 46.78 and 46.02, respectively, with Middle SES children scoring higher in the Dynamic Balance and Physical Competence domains, and High SES children leading in motivation and confidence and K&U domains. Significant differences were found between SES groups in PL and domain scores (p < 0.001), except for K&U (p = .958).

**Table 6:** Physical literacy and domain scores according to SES, STM, and location-wise.

Variable	PL Mean ± SD	DB Mean ± SD	PC Mean ± SD	K&U Mean ± SD	M&U Mean ± SD
**SES**					
Low SES (*N* = 906)	53.37±10.06	13.15±5.03	16.89±3.59	6.37±1.61	18.70±5.69
Middle SES (*N* = 261)	46.78±8.84	10.51±2.69	14.56±3.67	6.36±1.59	16.40±5.23
High SES (*N* = 193)	46.02±8.84	9.87±2.87	13.39±4.05	6.40±1.57	17.03±5.17
	<0.001	<0.001	<0.001	0.958	<0.001
**STM**					
Walk (*N* = 472)	55.15±10.79	13.98±5.81	17.45±3.24	6.62±1.62	19.48±5.47
Bicycle (*N* = 214)	50.04±8.63	12.02±3.84	16.01±3.81	6.11±1.54	17.47±5.61
Bike (*N* = 430)	49.58±9.22	11.35±3.32	15.48±3.95	6.23±1.59	17.25±5.50
School Bus (*N* = 177)	46.93±9.57	10.94±2.67	14.32±3.78	6.47±1.50	16.86±5.15
Car (*N* = 67)	45.90±7.91	10.03±3.36	12.47±4.29	6.18±1.59	17.45±5.32
	<0.001	<0.001	<0.001	<0.001	<0.001
**Residence Area**					
Urban (*N* = 1045)	50.27±9.62	11.86±4.31	15.65±3.80	6.40±1.60	17.79±5.44
Suburban (*N* = 315)	53.67±11.57	13.24±5.39	16.94±4.17	6.31±1.60	18.80±5.86
	<0.001	<0.001	<0.001	0.390	<0.005

**Note**: SES: Socioeconomic Status; STM: School Transport mode; DB: Daily Behavior; PC: Physical Competence; K&U: Knowledge & Understanding; M&C: Motivation & Confidence; PL: Physical Literacy; P-value < 0.001 means significant difference.

Children who walked to school had the highest mean PL scores and, in all domains, while those who cycled ranked second, except in K&U. The ’by car’ category had the lowest PL scores. Significant differences were observed across STM categories in PL domains (p < 0.001). Urban (n = 1045) and rural (n = 315) children differed in PL scores, with rural children scoring higher in PL (53.67) and in DB, PC, and M&C domains, but slightly lower in K&U (6.31) compared to urban children. Significant differences were noted between residential areas in PL and domain scores (p < 0.001), except for K&U (p = .390).

Table 7 revealed participant categorizations as per the CAPL-2 physical literacy stages. In the composite physical literacy scores, 71.6% (974 participants) were in the "Beginning" or "Progressing" categories, with 28.4% (386 participants) in "Achieving" or "Excelling." Among these, females predominantly occupied the lower categories (40% in "Beginning"/"Progressing"), while males were more represented in the higher categories (18% in "Achieving"/"Excelling").

**Table 7:** Gender-specific summary of participants among PL and domains interpretation categories of CPLA-2.

Variable	Total n (%)	Boys n (%)	Girls n (%)	*P*-value
**PL** (100 points)				
Beginning (< 41.99)	216 (15.9)	73 (5.4)	142 (10.4)	0.000
Progressing (42.00 to 55.00)	758 (55.7)	356 (26.2)	402 (29.6)	
Achieving (55.01 to 62.00)	217 (16.0)	131 (9.6)	86 (6.3)	
Excelling (> 62.00)	169 (12.4)	114 (8.4)	55 (4.0)	
**DB** (30 Points)				
Beginning (< 10.05)	767 (56.4)	310 (22.8)	457 (33.6)	0.000
Progressing (10.05 to 12.00)	332 (24.4)	214 (15.7)	118 (8.7)	
Achieving (12.00 to 13.86)	129 (9.5)	75 (5.5)	54 (4.0)	
Excelling (> 13.86)	131 (9.7)	75 (5.6)	56 (4.1)	
**PC** (30 points)				
Beginning (< 12.21)	251 (18.5)	63 (4.6)	188 (13.8)	0.000
Progressing (12.21 to 17.86)	680 (50.0)	322 (23.7)	358 (26.3)	
Achieving (17.87 to 20.00)	246 (18.1)	154 (11.3)	92 (6.8)	
Excelling (> 20.00)	183 (13.5)	136 (10.0)	47 (3.5)	
**K&U** (10 scores)				
Beginning (< 4.95)	216 (15.9)	93 (6.8)	123 (9.0)	<0.05
Progressing (4.95 to 6.93)	763 (56.1)	387 (28.5)	376 (27.6)	
Achieving (6.94 to 7.92)	264 (19.4)	145 (10.7)	119 (8.8)	
Excelling (> 7.92)	117 (8.6)	50 (3.7)	67 (4.9)	
**M&C** (30 scores)				
Beginning (< 12.90)	226 (16.6)	124 (9.1)	102 (7.5)	0.000
Progressing (12.90 to 20.89)	658 (44.8)	305 (22.4)	353 (26.0)	
Achieving (20.90 to 24.90)	312 (22.9)	143 (10.5)	169 (12.4)	
Excelling (> 24.90)	164 (12.1)	103 (7.6)	61 (4.5)	

**Note**: PA Behaviour: measured in the number of average weekly steps; Self-reported MVPA: measured in # of self-reported days;

In the daily behavior domain, 80.8% (1,099 participants) were classified as "Beginning" or "Progressing," and 19.2% (261 participants) as "Achieving" or "Excelling," with more females (42.3%) in the former and more males (11.1%) in the latter. For physical competence, 68.5% (931 participants) were in the lower categories, with a higher female representation (40.1%), while 31.6% (429 participants) in the higher categories saw more males (21.3%).

In the knowledge and understanding domain, 72% (979 participants) were "Beginning" or "Progressing," and 28% (381 participants) were "Achieving" or "Excelling," with a similar distribution between females (36.6%) and males (35.3%) in the lower categories, but slightly more males (14.4%) in the higher categories. In the motivation and confidence domain, 61.4% (884 participants) were in the lower categories, with females slightly more represented (36.5%), and 35% (476 participants) in the higher categories, with a marginally higher male representation (18.1%). Overall, most participants were classified as "Beginning" or "Progressing" across all domains, with a higher representation of females in these categories, except in "Achieving" or "Excelling" where males were slightly more represented.

### 3.1 Percentile curves

**Fig 4:**
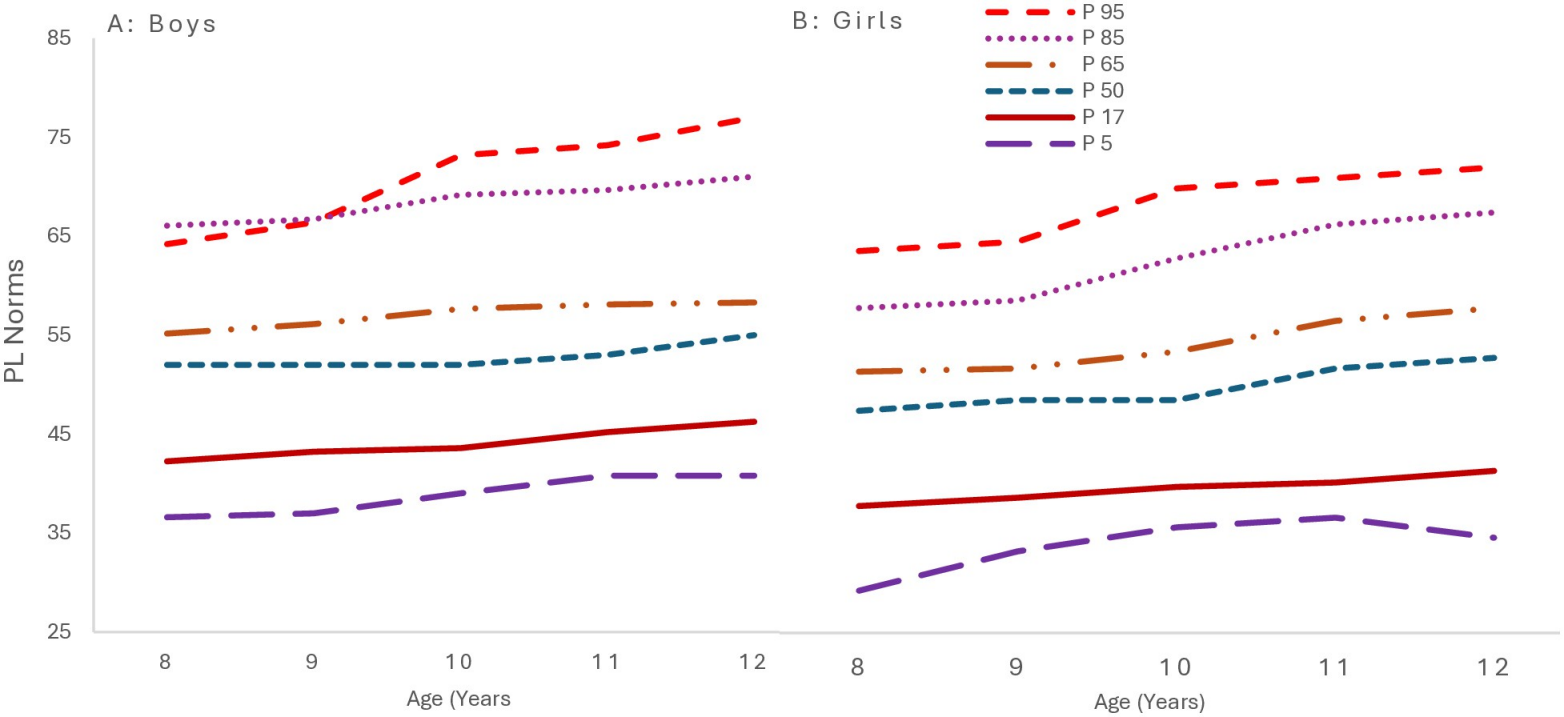
Smoothed percentile curves of physical literacy scores for 8-12-year-old children from South Punjab.

Fig 4 presents the GAMLSS-derived percentile growth curves. The growth curves indicate a steady rise in physical literacy as age increases for both genders. Notably, boys generally have higher percentile scores across age groups, although at age 12, girls’ P98 surpasses boys’ P90 and P75. These data reveal a consistent, albeit modest, annual increase in physical literacy for both genders, with boys slightly outpacing girls.

**Fig 5:**
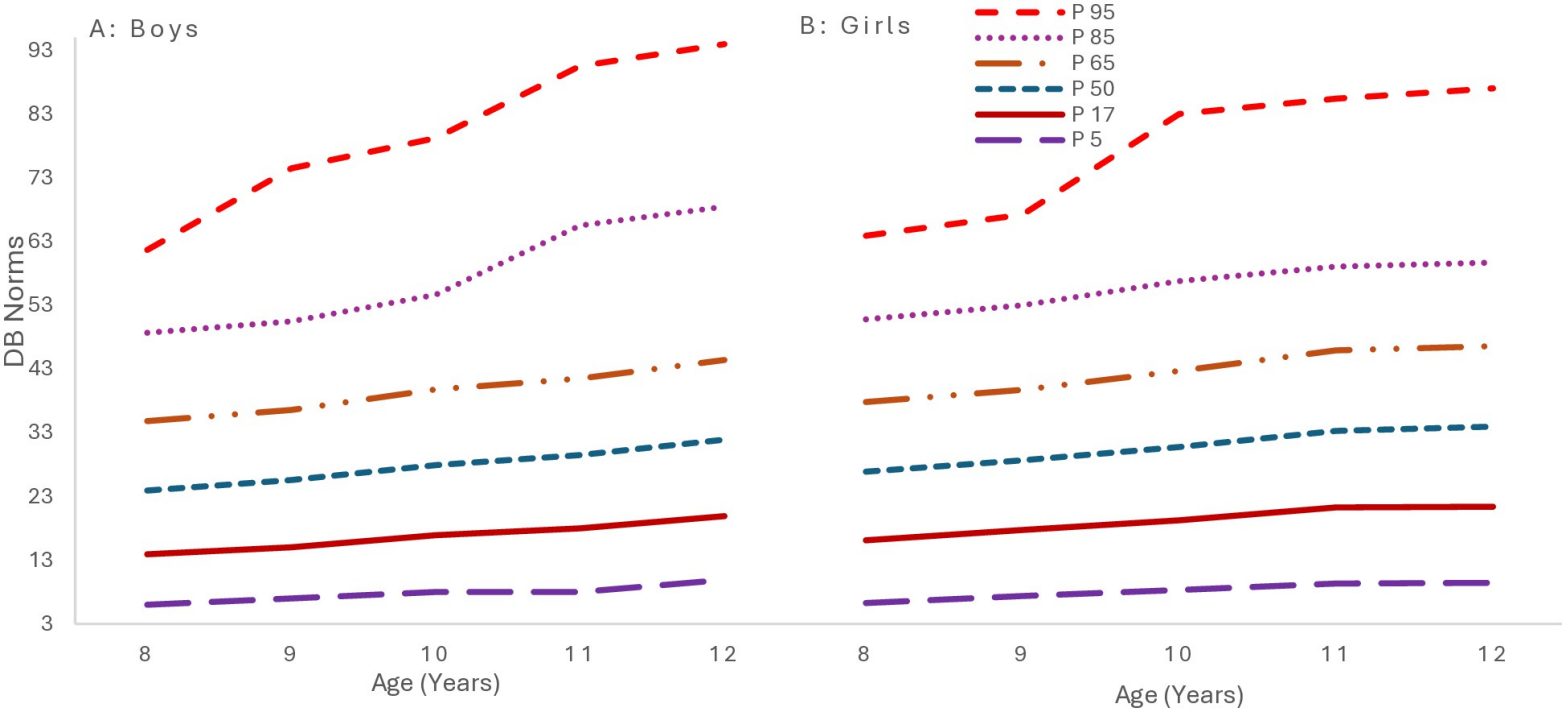
Smoothed percentile curves for daily behavior scores for 8-12-year-old children from South Punjab.

Fig 5 shows the GAMLSS-derived percentile curves. The growth curves reveal a decline in boys’ daily behavior as age increases, particularly evident in the P98 percentile, which exhibits a substantial increase. Conversely, the girls’ P98 percentile shows a less intense increase than those over same age range. The curves indicate that while both genders experience an increase in the range of daily behavior scores, the extent of this increase is notably higher in boys, especially at the upper percentiles.

**Fig 6:**
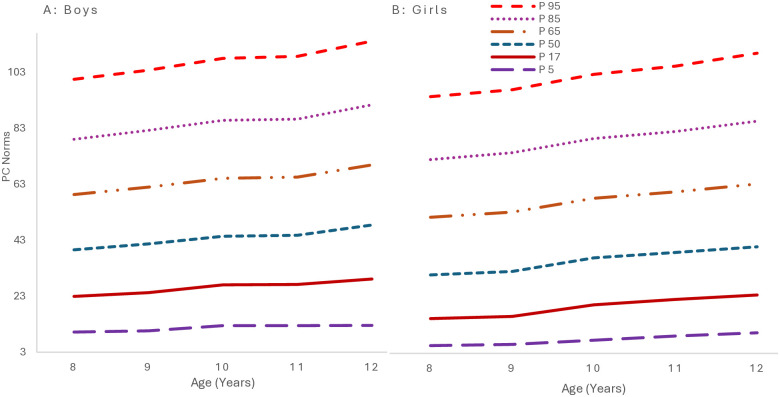
Smoothed percentile curves for physical competence scores for 8-12-year-old children from South Punjab.

Fig 6 presents the growth curves for the physical competence percentiles. The growth curves reflect a consistent enhancement in physical competence as age advances, with boys displaying a more pronounced progression across all percentiles. The annual increments highlight a continuous development in physical competence, with boys exhibiting a marginally greater yearly increase at the median percentile than girls.

**Fig 7:**
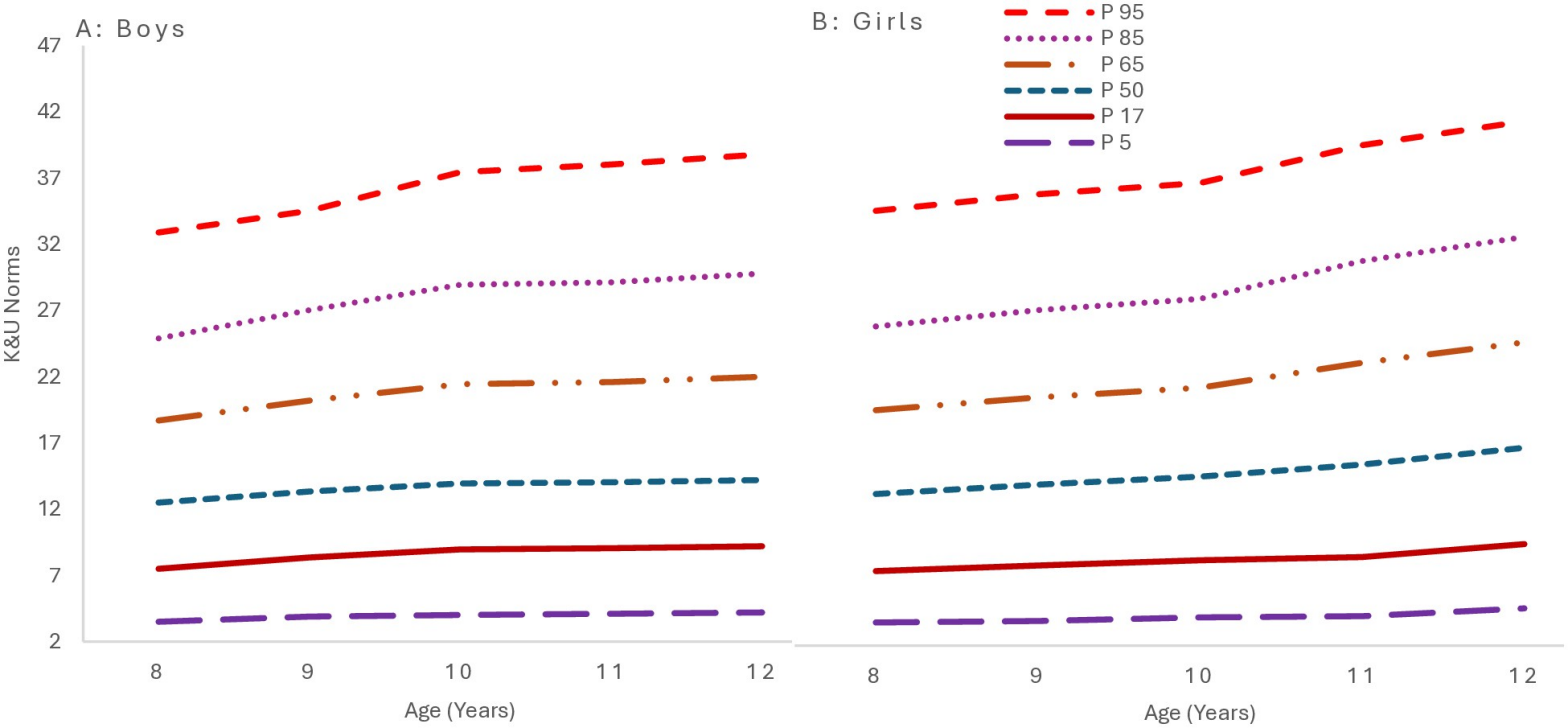
Smoothed percentile curves for K&U scores for 8-12-year-old children from South Punjab.

Fig 7 displays the Knowledge and Understanding domain GAMLSS-derived growth curves. Both boys and girls exhibit a consistent upward trajectory in median (P50) scores from ages 8 to 12. The highest percentile (P98) for both boys and girls also shows an upward trend, with boys’ scores rising. The growth curves corroborate a gradual increase in K&U scores with age, and the annual changes are consistent across genders.

**Fig 8:**
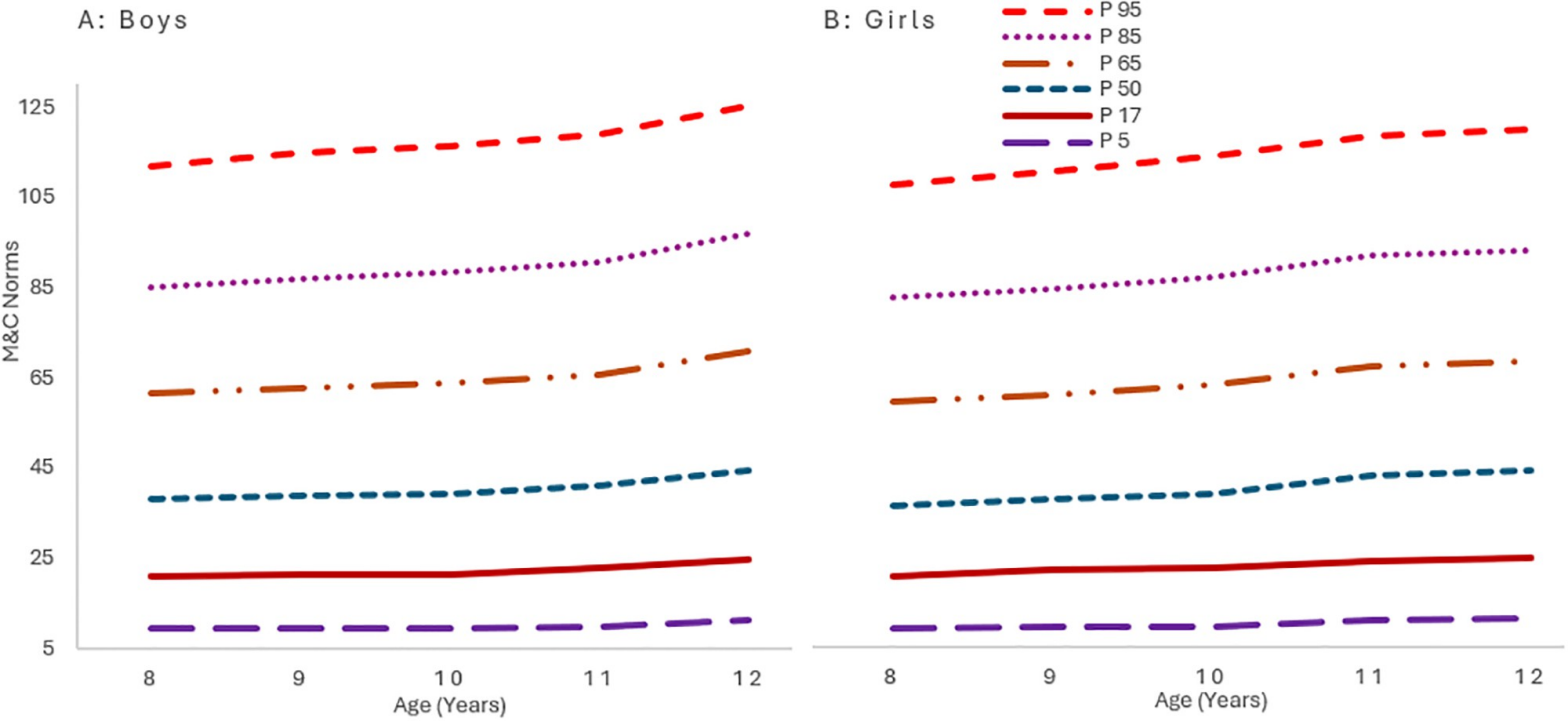
Smoothed percentile curves for M&C scores for 8-12-year-old children from South Punjab.

Fig 8 growth curves demonstrate a consistent elevation in M&C scores with advancing age. Boys exhibit slightly higher scores across all percentiles compared to girls, although the annual changes are nearly equivalent for both genders. These findings denote a steady, albeit slight, yearly enhancement in the M&C aspects of the children’s development.

Table 8 presented the correlations between Physical Literacy and its domain composite scores, as determined through Pearson correlation analysis. The results revealed a strong correlation between the PL Composite scores and three domains, namely Daily Behavior, Physical Competence, and Motivation & Confidence, with correlation coefficients (r) of 0.766, 0.785, and 0.844, respectively. Additionally, moderate correlations were observed between DB and PC (r = 0.581), DB and M&C (r = 0.513), and between PC and M&C (r = 0.450). On the other hand, the weakest correlations were detected between the total PL Composite scores and the Knowledge & Understanding domain, as well as between DB, PC and K&U, with corresponding correlation coefficients of 0.174, 0.021, and 0.032.

**Table 8:** Correlation among physical literacy and domains scores.

	**PL**	**DB**	**PC**	**K&U**	**M&C**
**PL**	1				
**DB**	.766**	1			
**PC**	.785**	.581**	1		
**K&U**	.174**	.021	.032	1	
**M&C**	.844**	.513**	.450**	.002	1

Note:

** Correlation is significant at the 0.01 level (2-tailed).

**Fig 9:**
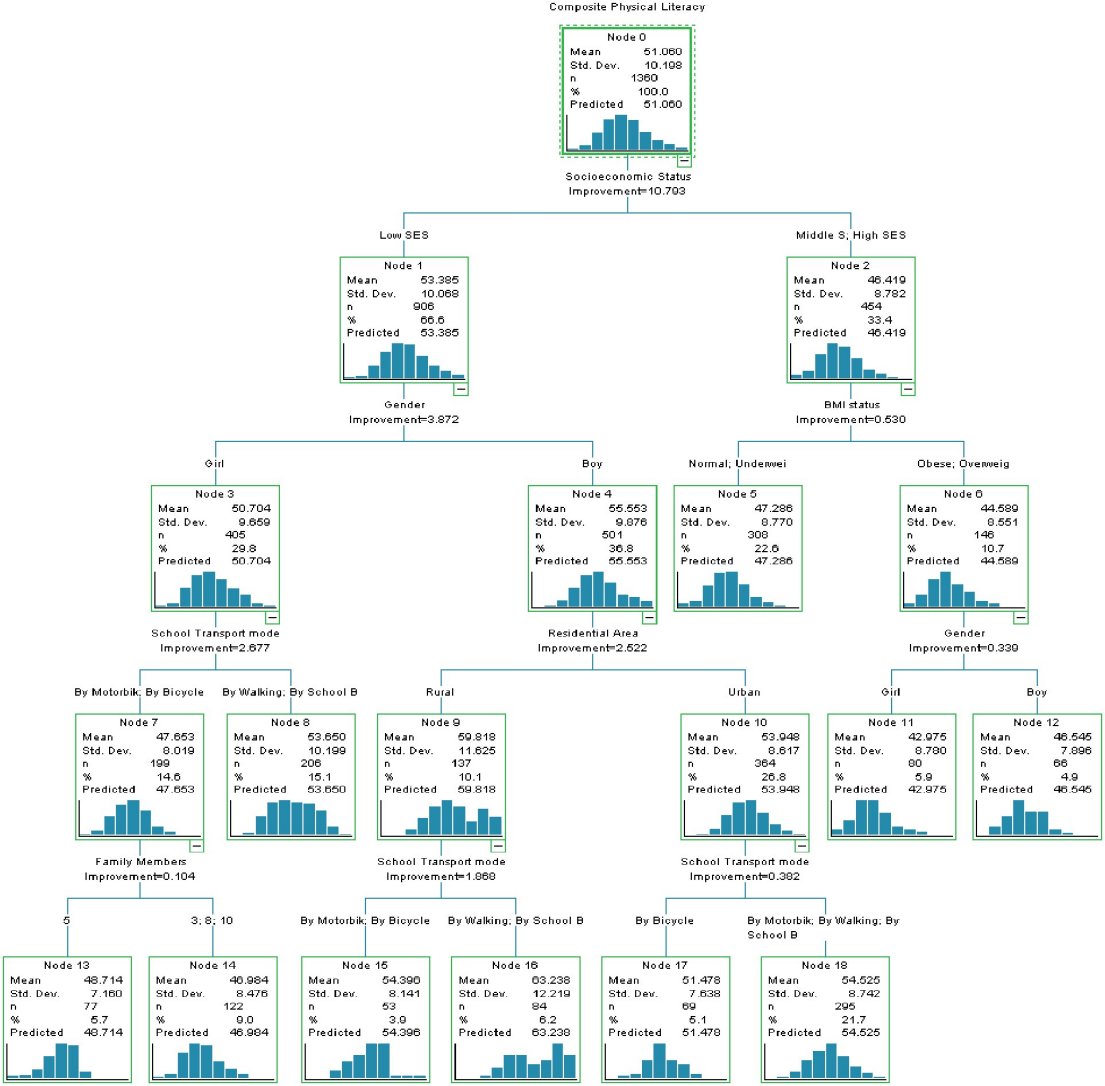
The decision tree with classification and regression tree (CRT) methods, highlighting the split criteria at each node.

Fig 9 shows the classification and regression tree diagram. Compared to the baseline model, which has an accuracy of 72.4%, the Decision Tree model demonstrates significant improvement. At the root node (Node 0), the mean composite physical literacy score is 51.060, with the first split based on socioeconomic status. Lower SES is associated with a lower mean score (Node 1: 53.385), while middle to high SES corresponds to higher mean scores (Node 2: 48.419).

Further down the tree, gender splits the low SES group, with girls having a mean score of 50.704 (Node 3) and boys 56.553 (Node 4). For the middle to high SES group, BMI status is the next differentiator, with normal to underweight individuals having a mean score of 47.286 (Node 5) and obese to overweight individuals scoring higher at 44.589 (Node 6). Subsequent splits are made based on residential areas and the mode of school transport. In rural areas, the mean score is 59.819 (Node 9), and in urban areas, it is 53.948 (Node 10). Different transport modes further divide these groups. For rural areas, transport by bicycle leads to the highest mean score of 61.478 (Node 17), while for urban areas, by motorbike/by walking/by school bus leads to a mean score of 54.625 (Node 18).

Overall, the decision tree suggests that SES, gender, BMI status, residential area, and school transport mode significantly influence composite physical literacy scores. Higher scores are more likely among boys, those from low SES backgrounds, rural residents, and students using bicycles as their mode of transport.

**Fig 10:**
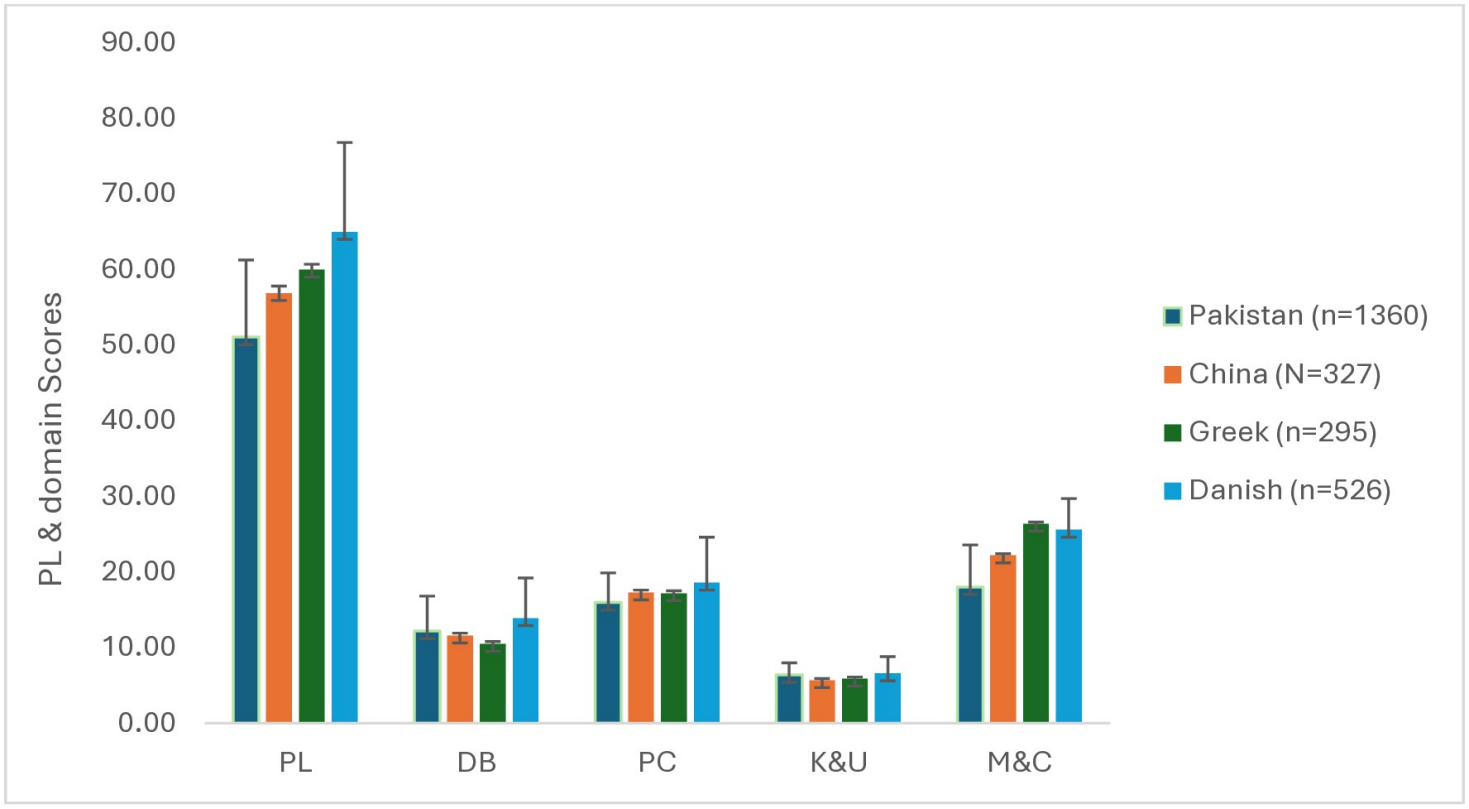
Country comparison of PL and domain scores. **Note**: PL: Physical Literacy; DB: Daily Behavior; PC: Physical Competence; K&U: Knowledge and Understanding; M&C: Motivation and Confidence.

Fig 10 illustrates a country comparison of mean physical literacy and domain scores based on CAPL-2 interpretive categories. Among the countries, the Danish study reported the highest physical literacy scores, followed by the Greek and Chinese studies, while the Pakistani study showed the lowest. In the daily behavior domain, the Danish study led with the highest scores, succeeded by the Pakistani and Chinese studies. Interestingly, the Greek study reported the lowest scores in this domain. As for the physical competence domain, the rankings were again led by the Danish study, followed by the Chinese and Greek studies, while the lowest scores were observed in the Pakistani study. The Danish study slightly edged out for the knowledge and understanding domain, followed by the Pakistani and Greek studies. The Chinese study, in this case, documented the lowest scores. As for the motivation and confidence domain, the Greek study stood out with the highest scores, succeeded by the Chinese and Danish studies. The Pakistani study again showed the lowest scores in this domain."

## 4 Discussion

The concept of physical literacy is increasingly gaining attention and necessitates future studies to track the trajectory of physical literacy in children and youth. This trajectory should be based on the primary components of PL, both physical and psychological, as proposed in widely accepted definitions. While these definitions have achieved consensus, empirical research has yet to validate them. In this context, the result of the present study provides the first comprehensive baseline assessment of the physical literacy status among school-age children aged 8 to 12 in South Punjab, Pakistan. The findings offer valuable insights into the influence of various demographic, socioeconomic, and environmental factors on physical literacy. The study also highlights associations between physical literacy domains (motor competence and psychological aspects) and physical activity, health-related fitness, and performance. It is important to note that emphasizing each PL domain and utilizing normative data to evaluate children’s PL in Pakistan is primarily intended to establish a foundational assessment. This assessment seeks to uncover key facets of PL that Pakistani children’s physical education or sports sectors have not adequately nurtured.

### 4.1 Demographics and physical characteristics

The study included participants from both genders, offering a comprehensive understanding of the physical literacy of boys and girls in South Punjab. The study covered the majority of South Punjab, with a significant portion of participants from urban areas (76.8%) of South Punjab aligns with the prevalent urbanization trend in developing nations, including Pakistan [38]. The participants’ physical characteristics, such as average height, weight, and BMI, provide a comprehensive picture of the children general health status, these findings align with the established growth standards for this age demographic in Pakistan [39].

However, a concerning trend emerged with 7.1% of participants being obese, with a marked skewness towards girls, reflecting global concerns about rising childhood obesity and subsequent health impacts [40]. Anthropometric measurements highlighted gender-specific variances; specifically, girls displayed higher values in hip circumference, body fat percentage, and overall body fat, whereas boys had heightened values in waist circumference and waist-to-hip ratio. Such variations are consistent with global data, emphasizing gender discrepancies in body composition during pre-puberty and puberty [41].

### 4.2 Socioeconomic and environmental factors

The study reveals that children from lower socioeconomic statuses in South Punjab display higher physical literacy scores than their higher SES peers. This contrasts with studies from developed countries, where higher SES often correlates with better health outcomes due to easy access to sports facilities, organized sports, and physical education, leading to higher physical literacy scores [42, 43]. The difference might be attributed to children from lower SES in developing countries, such as like South Punjab where children have more physically demanding daily routines or fewer sedentary distractions [44–46]. Another possible explanation could be the nature of daily activities and household tasks that children from lower SES backgrounds might engage in, which could contribute to their higher physical literacy.

Walking was identified as the major mode of transportation to school, implying a potential opportunity for increased daily physical activity and a direct association with higher physical literacy scores. Such findings are consistent with studies indicating that active commuting (e.g., walking, cycling) promotes physical health and contributes positively to overall physical literacy [47]. The significant association between gender and school transportation mode could indicate cultural or safety considerations specific to the south Punjab region, pointing to potential intervention strategies to encourage active commuting and consequently boost physical literacy.

### 4.3 Gender differences in physical literacy

The gender-specific scores in physical literacy and domains indicate significant gender differences. Boys consistently scored higher than girls in most physical literacy domains, except for Knowledge and Understanding, where the difference was nonsignificant. Such patterns align with a Chinese study where boys outperformed girls in all areas except K&U domain. However, when applied to the Pakistani context, current findings deviate, potentially due to socio-cultural factors favoring boys [10].

In Pakistan, boys’ benefit from greater exposure to sports and associated knowledge [48]; in contrast girls’ limited participation in sports might curtail their understanding of various health and fitness facets, emphasizing mainly on academics, resulting in a lack of knowledge regarding cardiovascular health, recommended physical activity, muscular endurance, and sports performance improvement [49]. This emphasizes the need to consider the sampled population since the CAPL-2 scoring is based on Canadian normative data [10]. Notably, our sample size surpassed other studies [10, 50, 51], and Pakistani boys and girls differ from their counterparts in other population studies, leading to score variations [10]. Future research should scrutinize the effects of sample size and demographics on assessment scores.

In Pakistani children, physical literacy significantly increases with age (P = 0.05). This trend is consistent across genders, with boys generally scoring higher [10, 51, 52]. Boys significantly outperformed girls in the domains of physical competence and daily behavior. Such outcomes align with Pakistani societal norms, which often permit boys more freedom for physical activities compared to girls [53, 54]. Further, the analysis regarding the status of DB indicates a trend in physical activity among boys and girls within a particular age range. Boys exhibit an increase in PA from ages 8 to 10, followed by a decrease at age 11, and then an increase again. Conversely, girls exhibit increased PA as they age, but a decline is observed at age 12. Overall, boys achieved higher scores than girls, and the results are similar to previous studies [50, 51].

Interpreting this trend in physical activity could have multiple interpretations, boys’ increased activity at ages 8–10 may be attributed to their interest in physical play and sports. The subsequent decline at age 11 might be linked to academic or social interests or limited opportunities for physical activity. Conversely, our study suggests that girls’ increased physical activity during this period is due to their interest and permission to play, as cultural and religious norms in Pakistan permit girls to participate in sports at this age [48]. However, the decrease in PA at age 12 is likely to be linked to puberty-related changes [55]. Cultural and religious norms and restrictions may limit girls’ sports and physical activities after puberty in Pakistan [56].

Within the physical competence domain, boys consistently outperformed girls, and domain scores increased as age advanced with significant (P >0.05) gender and age differences across various components. These findings align with other studies that have reported gender differences in specific physical skills and competencies [51, 57]. These differences could be attributed to biological factors, such as muscle mass and strength, or sociocultural factors, such as encouragement and opportunities provided to boys over girls in certain physical activities [48]. Incorporating developmentally suitable strategies and content within physical education curricula is imperative to facilitate the enhancement of movement skills and physical fitness among female students.

Based on the results, motivation and confidence domain scores were higher in boys than girls, with a significant difference (P = 0.05). Additionally, there was a noticeable trend in M&U scores concerning age. M&U scores in boys increased as they grew older, while girls’ M&U scores followed a different pattern. Girls’ M&U scores increased from ages 8 to 9, decreased at age 10, and then increased again from ages 11 to 12. The observed trend in boys’ M&U scores could be due to their participation in sports and physical activity. On the other hand, our study suggests that cultural and religious norms lead to reduced physical activity and sports involvement among girls as they approach puberty [48, 53]. As a result, girls’ M&C scores decrease as they grow older, a similar trend was observed in an Iranian study [58]. The data also demonstrates a higher level of motivation among girls for sports participation during their formative years. Inadequate motivation and a decline in self-confidence among female individuals are significant obstacles to their involvement in physical activities, as supported by several pertinent [59, 60].

However, the non-significant difference in the knowledge and understanding domain suggests that cognitive aspects of physical literacy are less influenced by gender [51]. Such findings are in line with global research that suggests biological differences, cultural norms, and access to physical activities can influence these outcomes [61]. The cultural context of South Punjab, where traditional gender roles might limit girls’ participation in physical activities, could further exacerbate these differences [53, 54]. The K&U scores showed a significant (P = 0.05) increase with age, consistent with previous research [10, 51]. Boys generally scored higher than girls, except for 12-year-olds, which contradicts the findings of a Greek study where only 8-year-old girls scored higher [51]. This difference warrants further investigation. Hence, there is a pressing need to design interventions that specifically address these gender-based challenges.

### 4.4 Status of physical literacy

Promoting physical literacy among children is essential for fostering a lifelong, physically active lifestyle [6]. This study, the first of its kind in South Punjab, Pakistan, aims to assess and raise awareness about the significance of physical literacy, paving the way for targeted interventions to realize the full potential of a healthy lifestyle. Utilizing validated measures, our results highlight that Children in South Punjab, regardless of age or gender, currently exhibit insufficient physical literacy levels. Despite their evident motivation to lead physically active lives, lack of knowledge and physical competence hinders their development of PL.

The findings indicate that most children, both boys and girls, are in the ’Beginning’ or ’Progressing’ stages of PL, underscoring an immediate need for intervention. This is in line with studies reported that children in various countries are at lower levels of physical literacy, attributed to factors such as limited sports infrastructure and the lack of emphasis on physical education in school curricula [62, 63]. An interesting gender disparity is observed, with girls (40%) having a higher representation than boys (31.6%) in these early stages. The result of the current study is similar to the Canadian [64, 65] and North American [66] studies, which identified that the children were at the progressing stage and highlighted a significant requirement for intervention. This finding confirms previous research indicating that PL development is an ongoing process during childhood and early adolescence, requiring consistent reinforcement and development [6].

A notable observation was the gender disparity in the physical literacy classification. Girls in this current study were mostly found in the "Beginning and Progressing" categories in all domains, a trend reflecting the broader societal norms and restrictions around physical activity for females in certain regions of Pakistan [54]. Such restrictions might reduce opportunities for girls to develop their physical skills and knowledge, resulting in the observed disparities [48]. Although not extensive, the boys did have a higher representation in the "Achieving and Excelling" categories. This can be associated with societal expectations and traditional roles that allow boys more freedom for outdoor activities, leading to better physical competence in Pakistan [48].

#### 4.4.1. Daily behavior

The most concerning result, perhaps, is the high percentage of children classified as "Beginning and Progressing" in the daily behavior domain, with 80.8% of participants falling into this category. This suggests that either the children may not be getting adequate opportunities for daily physical activity, or there may be a lack of knowledge regarding its importance [62]. This has potential implications for health, given the proven benefits of regular physical activity in childhood [67]. This emphasizes the importance of interventions aimed at promoting regular physical activity. The role of school programs and community-based interventions to instill a routine of daily physical activity becomes more evident from these results.

#### 4.4.2. Physical competence

Similarly, the findings within the physical competence domain, where 68.5% of the participants were categorized as "Beginning and Progressing", emphasize the need for structured physical education and sports programs in schools [68]. Regular exposure to varied physical activities can enhance children’s physical skills and motor development [69]. Interestingly, a higher proportion of girls were at the “Beginning and Progressing” stages compared to boys. This might be influenced by fewer opportunities for girls to participate in physical activities, lack of motivation, or even societal norms [48, 70].

#### 4.4.3. Knowledge and understanding

In the domains of knowledge and understanding, the gender distribution was relatively balanced, suggesting that while girls might have restricted physical activity opportunities, their knowledge and understanding towards physical literacy were not as hampered. This indicates that with the right interventions and opportunities, the existing gender disparities can be narrowed [6]. Notably, our data reveals a near-equal mean score between genders in the K&U domain of PL. This suggests that boys and girls exhibit comparable competencies in cognitive aspects of physical literacy, as supported by findings from Tremblay et al. [16].

#### 4.4.4. Motivation and confidence

Significant differences emerge between boys and girls upon examining the domain of motivation and confidence. Boys outperformed girls in three out of four components of this domain: predilection score, adequacy score, and intrinsic motivation score. These disparities were statistically significant, suggesting that boys in this sample displayed higher motivation and confidence in their physical abilities than girls. This disparity might stem from cultural and societal norms in South Punjab. Historically, boys often receive greater encouragement to engage in physical activities, whereas girls are frequently advised to remain at home for household learning or safety concerns [71, 72].

Furthermore, a significant proportion of children, 61.4%, were classified in the lower categories of "Beginning" or "Progressing" in the motivation and confidence domain. This suggests that a majority of the children in this region might not yet have developed the intrinsic motivation or confidence to engage in regular physical activity. Standage, Duda, & Ntoumanis (2003) emphasized that motivation is crucial for physical activity engagement, directly influencing physical literacy [73].

Our study’s findings emphasize the importance of interventions targeting daily physical activity, especially when a staggering 80.8% of participants fall into the initial categories. Moreover, the physical competence domain disparities suggest a need for structured physical education programs. At the same time, the relatively balanced scores in knowledge and understanding indicate that interventions can potentially bridge the existing gender gaps. As a roadmap, these results suggest educators, policymakers, and communities in regions like south Punjab must prioritize physical literacy tailored to address the unique socio-cultural challenges children face, particularly girls.

### 4.5 Impact of cofounding factors and correlations among PL and domains

For the first time, this study employed the decision tree model, particularly the classification and regression tree (CART) approach, to explore the impacts of multiple factors and their relationship with PL scores. This approach proved invaluable. When contrasted with the baseline model’s accuracy of 72.4%, the Decision Tree model exhibited a notable enhancement. Decision Trees are recognized for their interpretability and efficient handling of both numerical and categorical data.

The study identified Socioeconomic Status, Gender, and Weight Status as the critical features for predicting PL levels. Among these, Low SES emerged as the most influential predictor, with a PL importance score of 53.38. Previous research has highlighted the impact of socioeconomic differences on various health and educational outcomes. Children from lower SES backgrounds often encounter challenges accessing quality physical education, safe recreational areas, and essential resources, which can impede their physical literacy development [74]. Contrary to previous findings, this study revealed an inconsistency; children from lower SES backgrounds exhibited enhanced PL scores. Traditionally, lower SES has been correlated with poorer health behaviors, motor skills, and higher BMI [75]. The research proposes a potential reason for this inconsistency. It suggests that children from lower SES backgrounds in south Punjab, Pakistan, have higher physical activity scores in their daily behavior. This implies that they engage in more physical activities daily compared to their higher SES counterparts. In some cultures, or regions, children from low SES backgrounds are involved in more labor-intensive tasks or chores which require physical exertion, such as farming, transport mode, or assisting in household chores.

Following Low SES, the categories of Boys and Rural scored 55.55 and 59.81, respectively. This indicates that gender and living area also play a significant role in predicting PL levels. The results suggest that boys, especially those residing in rural areas, are predicted to achieve higher PL scores. This could be attributed to cultural norms and practices that might encourage boys to engage in outdoor activities more than girls, especially in rural settings [48, 71]. A noteworthy finding from the study is the impact of the Travel Mode to School on PL scores. The data suggests that rural children who either walk or cycle to school tend to score higher in PL. This is consistent with global research showing that active commuting to school, such as walking or cycling, is positively associated with children’s physical fitness and overall health [76].

The assessment of Physical Literacy among 8- to 12-year-old children from South Punjab, Pakistan, provides valuable insights into the relationships between the overall PL and its domains. A strong correlation was observed between the overall PL composite scores and three of its domains: daily behavior, physical competence, and motivation & confidence. Specifically, the correlation coefficients were 0.766, 0.785, and 0.844, respectively. These findings suggest that as children’s daily behaviors, physical competencies, and M&C levels improve, there is a concurrent and significant increase in their overall physical literacy. This is consistent with the foundational principles of physical literacy, which emphasize the importance of these domains in the holistic development of an individual’s physical literacy [77, 78]. Roetert et al. (2018) conclude that the association of these domains is crucial for engagement in physical activities throughout life [77].

Furthermore, moderate correlations were identified between the domains themselves. The correlation between DB and PC was 0.581, between DB and M&C was 0.513, and between PC and M&C was 0.450. This indicates that while these domains are distinct, they are interrelated. For instance, a child’s daily physical activities (DB) might influence their physical competencies and vice versa. Similarly, a child’s motivation and confidence can influence their daily physical behaviors and their physical competencies. This interconnectedness of domains underscores the importance of a multifaceted approach to enhancing physical literacy in children [79, 80].

However, it is noteworthy that the weakest correlations were found between the overall PL composite scores and the knowledge and understanding domain. The correlation coefficients for the relationships between the total PL composite scores and K&U, DB and K&U, and PC and K&U were 0.174, 0.021, and 0.032, respectively. This suggests that while knowledge and understanding of physical activity and health are essential, they might not be as strongly linked to a child’s overall physical literacy as the other domains. This finding resonates with previous research, which suggests that while knowledge is crucial, it alone might not be sufficient to drive behavioral changes in physical activity [51, 81].

The study highlights the significance of the daily behavior, physical competence, and M&C domains in the overall physical literacy of children from South Punjab, Pakistan. While K&U remains an essential domain, its weaker correlation with overall PL suggests that interventions aimed at improving physical literacy should prioritize enhancing DB, PC, and M&C levels while incorporating knowledge-based components. Future research should also consider the cultural and regional differences that might influence the development and understanding of physical literacy in diverse populations.

### 4.6 Percentile curves

The upward trend in physical literacy scores, as indicated by the median (P50) and highest percentile (P98) values, highlights the importance of age in the development of physical literacy [6]. Consistent with the findings of Whitehead (2010), this trend can be attributed to several factors associated with age, such as the natural development of motor skills, improved coordination, and a deeper understanding and engagement in physical activities [6]. As children grow, their physical capabilities and cognitive understanding of physical tasks typically enhance, contributing to higher physical literacy scores [82].

The observed gender differences, with boys generally outperforming girls in physical literacy scores, align with the research by Bremer et al. (2020) [83], which noted higher physical activity levels in boys. These differences could stem from a variety of factors, including societal norms that influence the types of activities encouraged for boys and girls, biological differences in physical development, and the availability of opportunities for engaging in physical activities [84]. In future longitudinal studies, exploring these factors in depth is crucial for understanding the root causes of these disparities.

The upward trend in physical literacy scores, evident in median (P50) and top percentile (P98) values, illustrates the significant role of age in physical literacy development [6]. This trend, consistent with Whitehead’s research, is attributed to age-related factors like motor skill evolution, enhanced coordination, and increased physical activity engagement. Children’s physical capacities and understanding of physical tasks progress with maturation, leading to improved literacy scores. Gender disparities are evident, with boys typically outperforming girls in physical literacy, aligning with Telford et al. (2022), who observed higher activity levels in boys [84]. These differences may stem from societal influences on gender-specific activities, biological developmental differences, and varying physical engagement opportunities. Future longitudinal studies should investigate these factors to understand gender disparities.

With age, cultural norms increasingly affect girls’ participation and performance in physical activities, possibly due to evolving interests and autonomy. The cultural sensitivity of PL assessments is crucial, as evidenced by adapting the CAPL-2 for Chinese populations [10]. Additionally, puberty’s physiological changes can distinctly affect physical literacy, differing genders, and influencing performance and interest in physical activities. Cairney et al. (2019) position PL as a determinant of health, linking it to motor coordination and the interplay between motor skills, physical activity, and health in children, suggesting that PL is influenced by various factors, including cultural and social environments [62].

Understanding the range of cultural influences on physical literacy, shaped by socioeconomic status, education, and regional differences, is critical. Insights into physical literacy dynamics inform educational and health policies, guiding interventions and programs to enhance literacy, especially in lower-performing groups [85]. Such understanding is crucial to fostering a healthier, more active population with long-term individual and public health benefits.

### 4.7 Country-wise comparison of physical literacy

The recent baseline assessment of the status of physical literacy among 8- to 12-year-old children from South Punjab, Pakistan, provides a unique insight into this region, especially when compared with findings from other countries. The country-wise comparison offers a comprehensive view of the mean physical literacy and domain scores based on CAPL-2 interpretive categories. The Danish study reported the highest scores across the studies accessing PL. This dominance could be attributed to Denmark’s robust physical education curriculum, early emphasis on physical activities, and a culture that promotes outdoor play. The Greek and Chinese studies followed, showing average scores in various PL domains. However, the Pakistani study falls behind, showing the lowest scores among the countries compared. This disparity might be indicative of the challenges faced by children in South Punjab, Pakistan, in terms of access to physical education, cultural barriers, or socio-economic constraints [86].

In the daily behavior domain, which assesses the patterns of children’s daily physical activity, the study from Denmark emerged with the highest average scores among all the studies examined. Following closely, the current study from Pakistan secured an impressive second place, surpassing the results from the Chinese study. This observation raises an interesting proposition: even with generally lower scores in physical literacy, children from South Punjab may be actively participating in daily physical routines. Such engagement could be influenced by cultural or environmental factors specific to the region [87]. Interestingly, students from lower socio-economic statuses recorded higher scores in this domain. Given that walking and cycling were the predominant modes of transportation to school among the study participants, this could potentially explain the higher scores.

Furthermore, the larger sample size in the current study might have influenced the overall results, suggesting the need for caution when comparing different studies. The Greek study’s lowest score in this domain is intriguing and warrants further investigation into Greek children’s daily routines and behaviors. The Danish study highlights leadership role, underscoring the importance Danish PE places on physical competence from a young age [50]. On the other hand, the Pakistani study’s lowest scores in the PC domain are concerning, highlighting potential gaps in physical education or opportunities for skill development in South Punjab.

### 4.8 Study strengths

This study is distinguished by its comprehensive methodology, which includes a large and diverse sample from South Punjab, Pakistan. The data collection was rigorously standardized using the validated CAPL-2 instruments and conducted by well-trained professionals, ensuring high reliability across the region [26]. Significant methodological strength also stems from the systematic and rigorous selection process, ensuring a representative demographic spread across diverse socioeconomic backgrounds, thus minimizing biases in the results [11, 26]. This research is pioneering in assessing physical literacy and establishing growth curves within a South Asian context, supported by a substantial participant base using the CAPL-2 tool.

Using pedometers to measure physical activity objectively adds value to the study by providing valid measured data. Nonetheless, it is crucial to recognize the inconsistencies when compared to accelerometers used in similar research. Future studies should explore the long-term effects of MVPA on PL and cardiorespiratory fitness to clarify causal relationships and assess the sustainability of these impacts. Further research should aim to identify which MVPA modalities most effectively enhance CRF and PL, potentially leading to more focused and impactful intervention strategies. This study establishes a foundation for understanding the relationship among activity, fitness, and literacy, which is crucial for the comprehensive physical development of children. Future investigations should delve into the barriers encountered by specific demographics, such as girls from low socioeconomic backgrounds, and develop strategies to overcome these obstacles. Additionally, longitudinal studies could provide insights into the long-term effects of these predictors on PL and overall health outcomes.

### 4.9 Implications

The findings from this study have several implications. Firstly, interventions aimed at improving PL should consider the children’s socioeconomic background. Efforts should be made to provide equal opportunities for physical activity and education for children from all SES backgrounds. Secondly, the gender disparity in PL scores highlights the need for targeted interventions for girls, especially in rural settings. Encouraging girls to participate in physical activities and sports can help bridge this gap. Lastly, promoting active commuting to school and active school breaks can be an effective strategy to enhance PL among children. Schools and communities can collaborate to create safe walking and cycling routes, thereby encouraging more children to adopt these modes of transport.

### 4.10 Limitations

While this study provides important insights, its interpretations are constrained by several limitations. The multicenter cross-sectional design prevents the establishment of causality between variables. To delineate these relationships longitudinally, future studies are needed. Additionally, the research, limited to specific geographical areas, may not extend to other socio-cultural contexts, highlighting the need for studies across diverse populations. Future investigations should also enhance structural equation modeling to incorporate all four domains of Physical Literacy, allowing for a more exhaustive analysis of the factors influencing PL.

## 5 Conclusion

This study offers a first assessment of physical literacy among 8- to 12-year-old children in South Punjab, Pakistan. The findings indicate a concerning low level of PL, with 71.6% of participants displaying early-stage PL. This aligns with similar international studies and highlights the pressing need for interventions, especially considering the evident gender disparities. Addressing these disparities, particularly the empowerment of girls, necessitates structured physical education programs and targeted strategies.

For the well-being of future generations in South Punjab, it is essential for educators, policymakers, and communities to prioritize PL. Tailored initiatives should address the specific socio-cultural challenges faced by children in this region. Furthermore, to deepen our understanding of PL and ensure its effective development, more research is needed. This should include expanding the existing database, validating the criteria used, and delving into the socio-cultural factors influencing PL in the Pakistani context. As this study provides a foundational framework, subsequent research should incorporate qualitative methods to better understand the barriers and facilitators of PL, promoting a contextually relevant approach for its enhancement in South Punjab, Pakistan.
